# Impact of Different Application Parameters of Cold Atmospheric Plasma on Foodborne Pathogen Inactivation

**DOI:** 10.1002/jemt.24838

**Published:** 2025-02-27

**Authors:** Berat Cinar Acar

**Affiliations:** ^1^ Faculty of Science, Department of Biology Gazi University Ankara Turkiye

**Keywords:** cold atmospheric plasma, food quality, food safety, foodborne pathogens, preventing microbial growth

## Abstract

Foodborne pathogens are a major public health concern, causing millions of illnesses and deaths yearly. Traditional thermal processing methods, such as cooking and pasteurization, are effective at killing pathogens, but they can also damage food quality. Cold atmospheric plasma (CAP) can inactivate foodborne pathogens without damaging food quality. CAP has several advantages over traditional thermal processing methods. It is a non‐thermal process, meaning that it does not heat food. This can help to preserve the nutritional and sensory quality of food. CAP can be targeted to specific areas of food, such as the surface or interior. This can help to reduce the overall processing time and energy consumption. In the study, two Gram (+) ( *Staphylococcus aureus* ATCC 25923, *Listeria monocytogenes* ATCC 7644), three Gram (−) ( *Salmonella typhimurium* CCM 5445, *Salmonella enteridis* ATCC 13076, *Escherichia coli* O157:H7), and one yeast culture ( *Candida albicans* ATCC 10231), known as pathogens, were used to examine the influence of CAP on microorganisms. The samples were treated with CAP at different power rates (100, 150, and 200 W) and exposure times (30, 60, 180, and 300 s) with different application parameters (directly to microorganisms, distilled water, microorganism + distilled water combination). Then, the number of viable cells was determined after the procedure. Among the methods, it was found that the direct cold plasma application is the most effective for the inhibition of microorganisms. Besides, it was designated that the inhibition of pathogen microorganisms increased as the power rate and contact time enhanced. Cold plasma treatment induced membrane damage in microorganism cells, with the severity of damage increased with longer treatment times. A 300‐s direct plasma exposure induced cell lysis and membrane disintegration, highlighting the potential of this technology. This study aimed to investigate the potential of CAP technology to control microbial contamination in food and agriculture, focusing on determining optimal treatment parameters and understanding the morphological changes induced in bacteria by the plasma.

## Graphical abstract

The study examines the potential of CAP technology as a means of preventing microbial contamination in food and agricultural products. Applying the CAP system with different parameters (duration, power) and methods (microorganism, distilled water, and microorganism+distilled water combination) results in the generation of ROS and RNS, which exhibit inhibitory effects on pathogenic microorganisms.

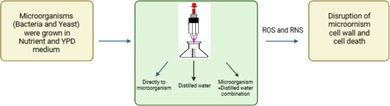

**Summary**

- Cold plasma is an innovative technology with excellent potential in food and agriculture.
- Direct cold atmospheric plasma (CAP) application was the most effective method for microbial inhibition.
- CAP is more effective at inhibiting the proliferation of pathogenic bacteria compared to yeast.
- Increasing the power and contact time of treatment further enhanced the microbial inhibition effect.

## 1 Introduction

Plasma, essentially an ionized gas, is composed of various charged particles, including electrons, positive and negative ions, excited atoms and molecules, radicals, metastables, and vacuum ultraviolet and ultraviolet photons (Malyavko et al. 2020; Simeckova et al. 2020; Ansari et al. 2021). Gas and enough energy to ionize the gas are needed to create plasma. Although plasma consists of charged species, it is generally neutral (Busco et al. 2020). Plasma systems are categorized according to their thermodynamic properties (high and low temperature) and operating pressures (low and atmospheric pressure). Thermal plasmas are produced by heating gas at high temperatures and under conditions where electrons and ion species are in thermodynamic equilibrium. Their temperature is several thousand Kelvin at atmospheric pressure (Xu et al. 2020). Non‐thermal (cold) plasmas are characterized by ions and mobile molecules that contain high energy at low temperatures and are not thermodynamically stable (Bourke et al. 2018).

Cold plasma is a distinctive type of plasma at a temperature below 40°C at the application site (Kandemir et al. 2021). Cold plasmas are produced primarily through the energy from alternating or direct electrical currents, radio frequencies, or microwaves. Various configurations, such as volume and surface dielectric barrier discharge (DBD), atmospheric pressure plasma jets, plasma needles, and plasma pens, are used to produce these ionized gases. The gas mixtures used may include normal atmospheric gases such as Oxygen (O_2_), Ozone (O_3_), Nitrogen (N_2_), Hydrogen (H), and Carbon Dioxide (CO_2_), as well as noble gases such as Helium (He) and Argon (Ar) (Han et al. 2020a).

Plasma technology applications can be performed on various types of solid or liquid targets. Particular attention is paid to plasma interactions within and with liquids (Bruggeman et al. 2016). Typical applications of plasma in this field focus on wastewater treatment (Kozáková et al. 2010), reforming of hydrocarbons (Xin et al. 2020), production and purification of nanoparticles (Sharma et al. 2017; Kozáková et al. 2018), and surface treatments of solid materials (Klíma et al. 2006; Kravets et al. 2015). Cold plasma technology stands out as a promising alternative for modifying the properties, including the quality and composition of various materials, such as water, oil, buffer solutions, soil, gases, seeds, plants, microbes, viruses, and even the surfaces of objects (Chaplot et al. 2019; Misra et al. 2019; Yadav and Roopesh 2020; Gao et al. 2022).

Rapid population growth, dwindling agricultural land, climate change, and escalating water scarcity pose significant challenges to modern agriculture and the food industry. These challenges, coupled with the presence of bacterial pathogens, foodborne viruses, bacterial toxins, pesticide residues, and mycotoxins, contribute to critical food and agricultural safety concerns (Bourke et al. 2017). Foodborne pathogens, the primary cause of foodborne illnesses, represent a major global health challenge (Elbehiry et al. 2023). These pathogens, encompassing bacteria, viruses, parasites, and fungi, contaminate food sources and can cause a range of illnesses in humans. Originating from diverse sources such as animals, humans, soil, and water, foodborne pathogens are responsible for numerous infections, impacting both human health and the economy through their adverse health consequences (Moi et al. 2022). A diverse array of pathogenic microorganisms can contaminate food products throughout the entire food chain, from production and processing to storage and distribution (Carstens et al. 2019; Moi et al. 2022). Consumption of contaminated food can lead to a range of symptoms, including stomach aches, diarrhea, abdominal pain, fever, and muscle aches. The severity of illness can vary, with some cases being mild and others more severe (Belina et al. 2021; van Puyvelde et al. 2021). Foodborne pathogens pose a significant risk to human health, particularly affecting vulnerable populations such as children, the elderly, and individuals with weakened immune systems (Cherif et al. 2023).

Microbial contamination, which poses a growing critical hazard to global public health, negatively affects the nutritional values, color, and edibility of agricultural and food products and can cause epidemics and severe economic losses (Amit et al. 2017). Therefore, it is crucial to use appropriate processing methods to provide the microbiological safety of the products. Food processing can be achieved using various techniques like pasteurization, high‐pressure processing, distillation, ozonation, irradiation, ultrasonication, and chemical treatments (Waskow et al. 2018; Rana et al. 2020). However, such processes may damage food quality, toxicology, and nutritional properties (Ozen and Singh 2020; Domonkos et al. 2021). To meet the growing demand for safe and fresh food, scientists have developed various non‐thermal preservation techniques, including ultrasound, high‐pressure processing, UV treatment, pulsed electric field treatment, cold plasma, irradiation, and electrolyzed water (Liao et al. 2017). Among these techniques, cold plasma has emerged as a powerful tool for decontaminating food products (Sahoo et al. 2023). Cold plasma has become a topic in food and agriculture, with its ability to disinfect food and improve its longevity, capturing widespread attention (Hernandez‐Hernandez et al. 2019). With the increasing consumer demand for fresh, safe, high‐quality, and nutritious agricultural and food products, CAP technology has been comprehensively studied (Dasan and Boyaci 2018; Hernandez‐Hernandez et al. 2019; Chen et al. 2020). CAP has the potential to be effective against bacteria, viruses, mycotoxins, and yeasts in plant and animal‐based foods. Agricultural products are frequently contaminated during the harvest and post‐harvest (transportation, storage, cleaning, packaging, and food processing) stages due to contact with dust, insects, animal urine and feces, workers, and equipment (Sakudo et al. 2019; Adhikari et al. 2020). CAP applications can potentially inactivate bacterial and fungal pathogens that cause contamination in agricultural products.

The efficacy of plasma treatment is contingent upon various factors, including treatment time, voltage, gas composition, product type, microorganism type, and food surface characteristics (Xiang et al. 2018; Niveditha et al. 2021). The precise mechanisms underlying plasma sterilization are not fully understood due to the complex interactions between plasma species and microorganisms. However, numerous studies have demonstrated that UV radiation and charged particles generated during plasma ionization contribute significantly to microbial inactivation (Gaunt et al. 2006; Das et al. 2022; Rao et al. 2023). Plasma‐generated reactive species, including reactive oxygen species (ROS) and reactive nitrogen species (RNS), oxidize lipids and sugars in microbial cell membranes, resulting in cell lysis (Yusupov et al. 2013). These species can further penetrate the cell wall, disrupting peptidoglycan bonds and damaging vital biomolecules like DNA and proteins (Misra and Jo 2017). The intensity of UV radiation emitted during plasma generation, which can induce DNA damage and inhibit bacterial growth, is influenced by the specific plasma device and gas composition (Eto et al. 2008).

In addition to plasma technology, plasma‐activated water (PAW) applications can also be used to control plant diseases by inactivating pathogens (Hertwig et al. 2018; Adhikari et al. 2020; Feizollahi et al. 2020; Tamošiūnė et al. 2020). CAP technology is shown as an alternative to traditional methods due to its non‐thermal nature, improving microbiological safety and preserving the quality characteristics of a wide range of foods within rapid processing times and sterilization, ease of application, low operating costs, and environmental friendliness (Bourke et al. 2017).

This research aimed to evaluate the potential of CAP technology for preventing microbial contamination in food and agricultural products. Therefore, a plasma system consisting of two conductive electrodes, a gas inlet placed between these electrodes, and an electrical voltage input was used. It was aimed to investigate the bactericidal/bacteriostatic effects of CAP on pathogenic microorganisms by applying different parameters (application time and power) and methods (bacteria, distilled water and bacteria + distilled water combination) and also aimed to identify the specific morphological changes in the bacteria that experienced the highest level of inactivation.

## 2 Methodology

In this study, Gram (+) *Staphylococcus aureus* ATCC 25923, *Listeria monocytogenes* ATCC 7644, Gram (−) *Salmonella typhimurium* CCM 5445, *Salmonella enteridis* ATCC 13076, *Escherichia coli* O157:H7, and *Candida albicans* ATCC 10231 yeast, which were in the stock culture collection of Gazi University, Faculty of Science, Department of Biology, Biotechnology Laboratory, were used.

Nutrient (Merck) and YPD (Yeast Extract‐Peptone‐Dextrose, Merck) medium was used as a liquid medium to grow the bacteria and yeast, respectively. For inoculum build‐up of microorganisms, 2% of samples were inoculated in a liquid medium and incubated at 37°C for 24 h. Then, to determine live cell counts, *S. aureus* ATCC 25923 (Mannitol Salt Phenol Red Agar, Merck), *L. monocytogenes* ATCC 7644 (Listeria Enrichment Agar, Merck), *S. typhimurium* CCM 5445 and *S. enteridis* ATCC 13076 (Salmonella Shigella Agar, Merck) and *E. coli* O157:H7 (Eosin Methylene Blue Agar, Merck), and *C. albicans* ATCC 10231 (YPD Agar, Merck) were inoculated in selective nutrient media and incubated at 37°C for 24 h after CAP application (Rusenova et al. 2022; Tavassoli et al. 2022; Sadeq et al. 2024).

### 2.1 Properties of CAP

**FIGURE 1:**
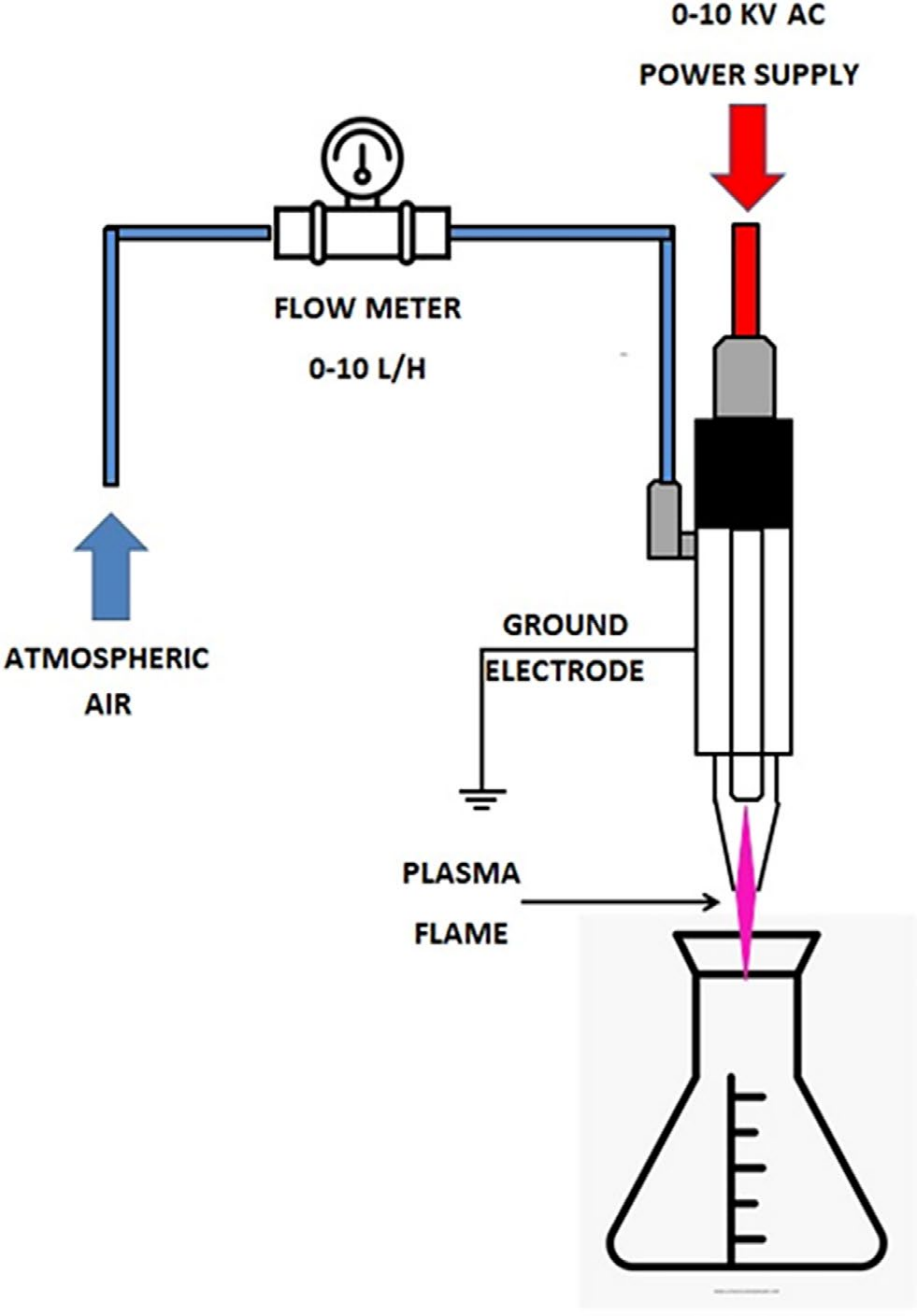
A diagram of an atmospheric plasma jet (Drawn with BioRender).

The study used a cold plasma system under atmospheric air supplied by a 600 W AC atmospheric plasma jet with 0–10 KV (Monoplasma, MP600). The experimental setup of the CAP system used in the study is shown in Figure 1.

### 2.2 CAP Application to Microorganisms

**FIGURE 2:**
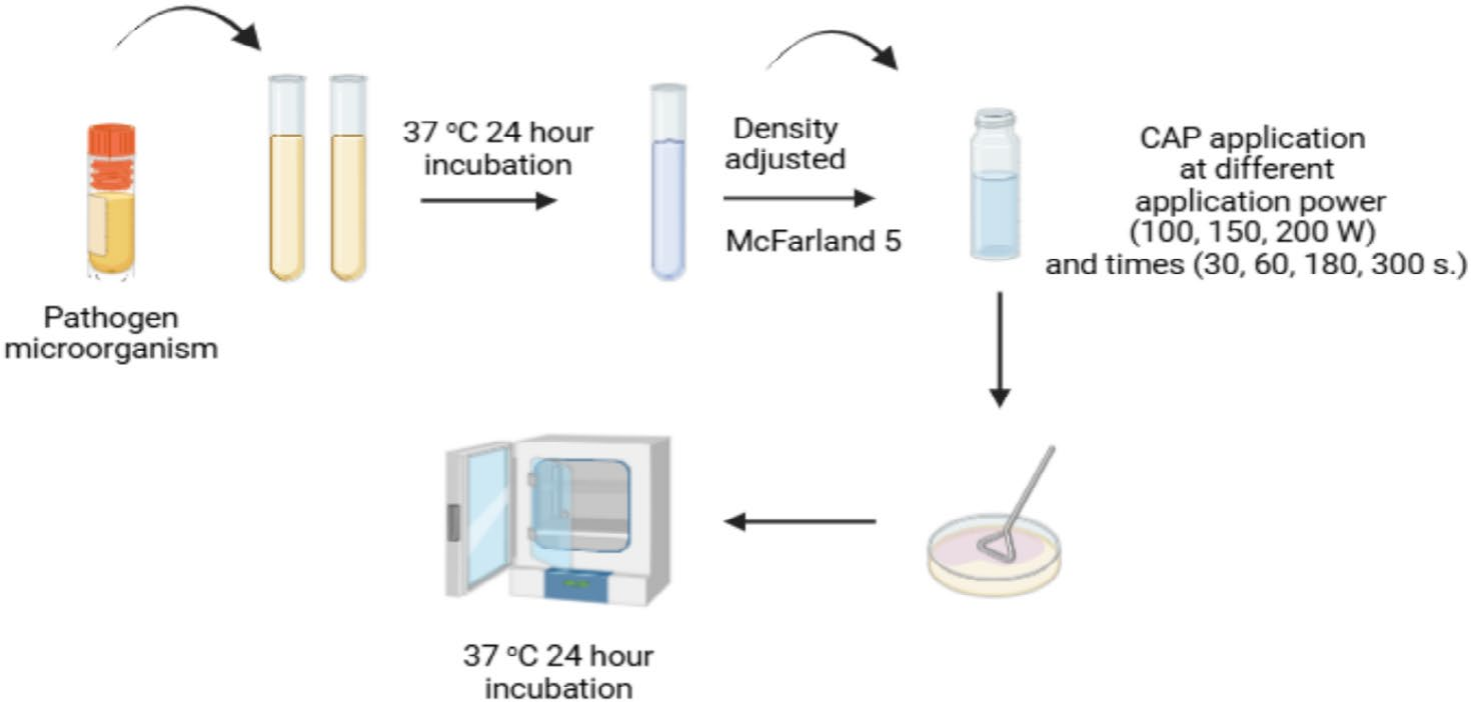
CAP application to microorganisms (Drawn with BioRender).

The Gram (−) and Gram (+) pathogenic microorganisms were grown in the Nutrient (Merck) medium, and *C. albicans* were grown in the YPD (Merck) medium. The optical densities of bacteria and yeast cultures were adjusted to McFarland 5 (1.5 × 10^9^ log CFU/mL). Then, CAP was applied at different times (30, 60, 180, and 300 s.) and application powers (100, 150, and 200 W). A 100 μL of the microorganism was taken, cultivated on the suitable solid medium for each microorganism, and incubated at 37°C for 24 h (Figure 2).

Colonies were calculated according to the formula below.

$$\begin{array}{c} \text{Number of Live Bacteria}\;\left(\mathrm{CFU}/\mathrm{mL}\right)=\\ \left(\text{Number of colonies}\times \text{Dilution factor}\right)/\\ \text{Bacteria inoculated in the Petri dish}\;\left(\mathrm{mL}\right) \end{array}$$

Dilution factor=1/Dilution ratio

### 2.3 CAP Application to Water Sample

**FIGURE 3:**
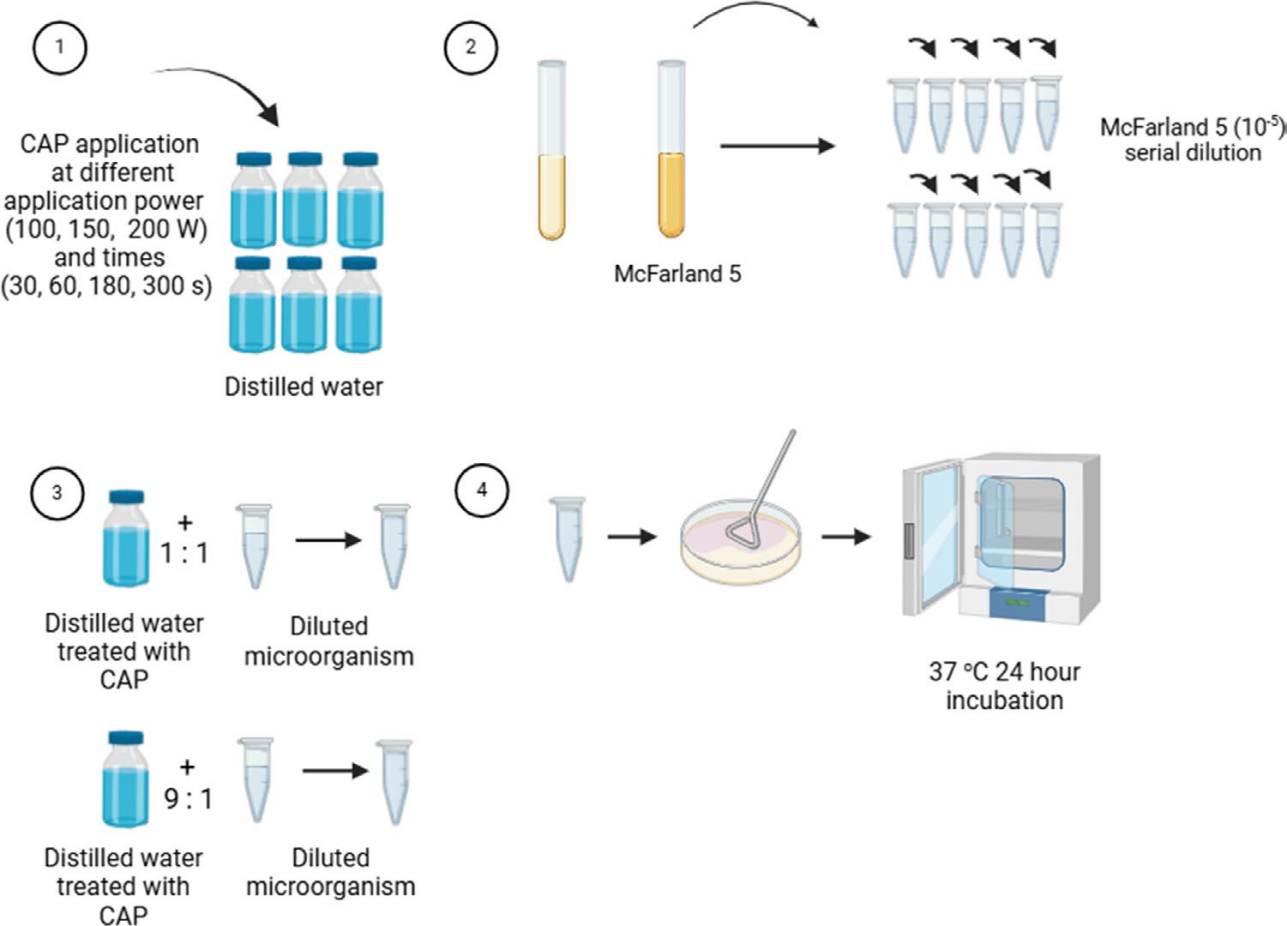
CAP application to distilled water (Drawn with BioRender).

Distilled water (100 mL) was treated with CAP at different times (30, 60, 180, and 300 s.) and application power (100, 150, and 200 W). Microorganisms' densities were adjusted to McFarland 5, and samples of microorganisms and distilled water were taken at ratios of 1:1 (1 mL microorganism and 1 mL CAP distilled water) and 1:9 (1 mL microorganism and 9 mL CAP distilled water) and mixed until homogeneous. For live colony counting, 100 μL of the total mixture was taken and inoculated on a suitable solid medium for each microorganism. It was then incubated at 37°C for 24 h (Figure 3).

### 2.4 CAP Application to Distilled Water + Microorganisms Combination

**FIGURE 4:**
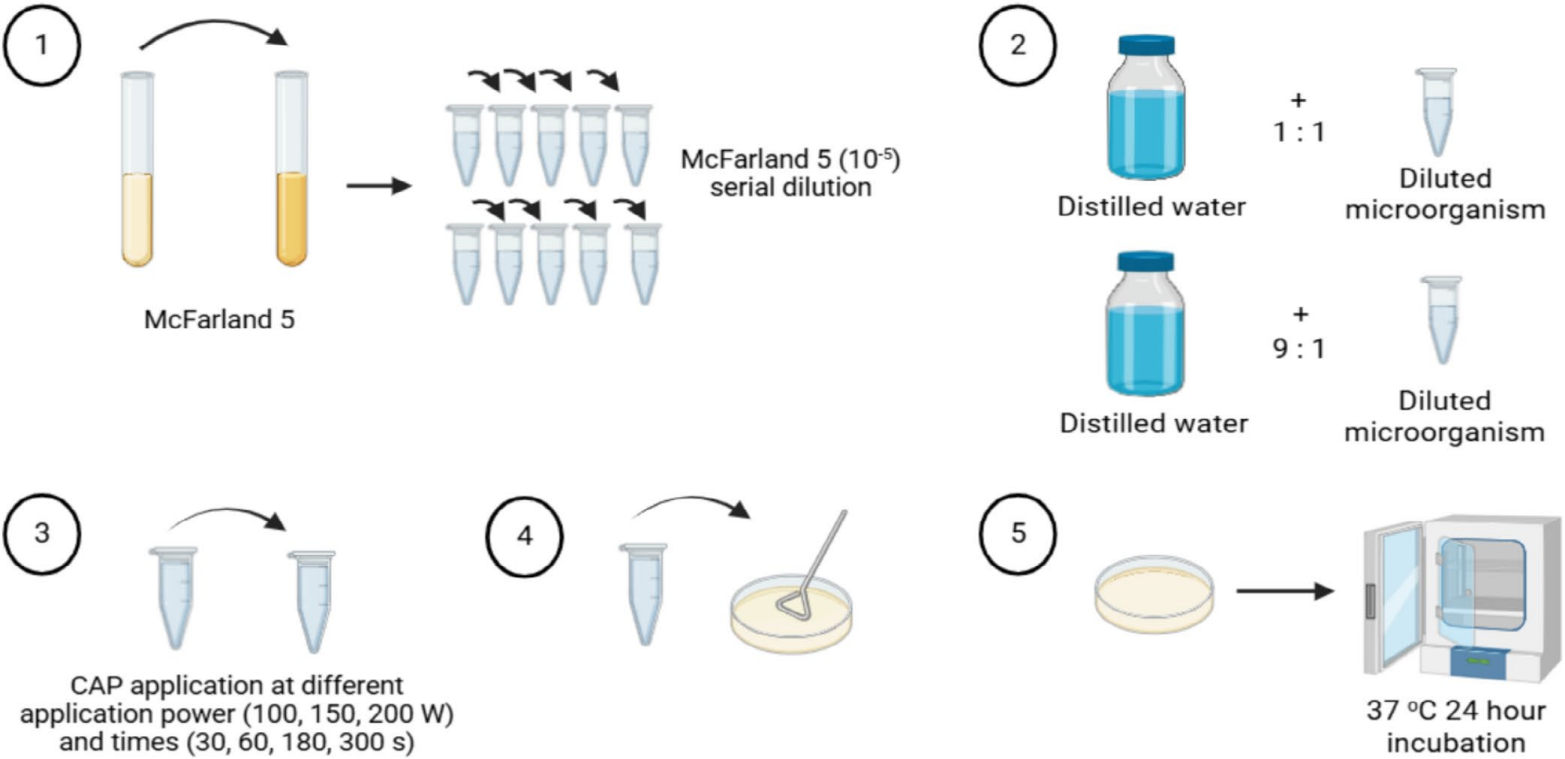
CAP application to distilled water + microorganisms combination (Drawn with BioRender).

Microorganism densities were adjusted to McFarland 5, and samples were taken from microorganisms and distilled water in ratios of 1:1 (1 mL microorganism and 1 mL distilled water) and 1:9 (1 mL microorganism and 9 mL distilled water). Then the solution was mixed until it became homogeneous. Then, samples were treated with CAP at different times (30, 60, 180, and 300 s.) and application power (100, 150, and 200 W). Cultivation was carried out on solid media specific to each bacterium for live cell counting. After inoculating in a petri dish, the samples were incubated at 37°C for 24 h (Figure 4).

### 2.5 Scanning Electron Microscopy Analysis

The changes in the surface structure of *E. coli* bacteria, which showed the highest inactivation after CAP treatment, were examined using scanning electron microscopy (SEM) analysis. The SEM images were acquired using a Quanta FEG 250 FEI. The SEM analysis was conducted at the ODTU MERLAB, Türkiye.

### 2.6 Statistical Analysis

All studies were carried out in three parallel, and the average results of the values were given. Data obtained from these studies were presented as the mean of these replicates ± Standard Deviation (SD). SPSS Inc. Software (version 22.0, SPSS Inc., Chicago, IL) was used in statistical analyses. Whether there was a significant relationship between CAP applied at different application powers and times and the number of living cells was detected by one‐way ANOVA analysis, Tukey's post hoc test, and using GraphPad Prism (www.graphpad.com) software. Statistical significance values were set at *p* < 0.05.

## 3 Results

This study investigated the effectiveness of CAP in inhibiting microorganisms. Microorganisms were exposed to CAP at varying power levels (100, 150, and 200 W) and times (30, 60, 180, and 300 s.). Remarkably, no microorganism growth was observed at any power level at longer treatment times (180 and 300 s.), indicating complete elimination. Despite CAP treatment for 30 or 60 s, microorganisms could still be detected, though at a lower level than in the control group (microorganisms without CAP application) (Tables 1 and 2). Among the microorganisms studied, *C. albicans* ATCC 10231 exhibited the smallest reduction in viable cell count (6.71%, 7.24 log CFU/mL) after 30‐s exposure to CAP at 100 W, which was the shortest treatment duration employed. The highest decrease in viable cell count was observed in *E. coli* O157:H7, with a reduction rate of 60.80% (3.16 log CFU/mL) at 60 s and 200 W (*p* < 0.05).

**TABLE 1:** Number of live cells (log CFU/mL) as a result of CAP application to microorganisms.

Microorganism name	100 W				150 W				200 W				Control micoorganism log CFU/mL
	Time (s)												
	30 s^a^	60 s^x^	180 s	300 s	30 s^b^	60 s^y^	180 s^y^	300 s	30s^c^	60 s^z^	180 s	300 s	
*S. aureus* ATCC 25923	7.43 ± 1.9^z^	6.35 ± 2.7	—	—	7.14 ± 2.1	5.37 ± 0.8	—	—	6.69 ± 1.1	4.44 ± 1.6^a^	—	—	8. 15 ± 1.4
*L. monocytogenes* ATCC 7644	7.42 ± 1.3^z^	6.18 ± 2.2	—	—	6.93 ± 1.2^z^	5.20 ± 3.0	—	—	6.56 ± 2.4	4.02 ± 2.0^a,b^	—	—	8.21 ± 1.7
*S. typhimurium* CCM 5445	7.06 ± 2.1^z^	5.65 ± 1.7	—	—	6.54 ± 2.3^z^	4.76 ± 1.1	—	—	5.97 ± 0.9	3.59 ± 1.4^a,b^	—	—	7.92 ± 1.1
*S. enteritidis* ATCC 13076	7.18 ± 1.9^z^	5.69 ± 2.5	—	—	6.53 ± 1.7^z^	4.75 ± 1.3	—	—	6.21 ± 2.6	3.52 ± 2.2^a,b^	—	—	8.14 ± 2.5
*E. coli* O157:H7	6.99 ± 1.2^z^	5.27 ± 1.7	—	—	6.46 ± 2.8^z^	4.32 ± 2.1	—	—	5.87 ± 1.6	3.16 ± 1.2^a,b^	—	—	8.05 ± 1.6
*C. albicans* ATCC 10231	7.24 ± 2.0	6.59 ± 1.4	—	—	6.95 ± 1.7	6.12 ± 1.3	—	—	6.39 ± 2.2	4.64 ± 3.1	—	—	7.76. ± 0.9

*Note:* —: No reproduction observed. ±: presented as standard deviation. ^a,b,cx,y,z^Values expressed with different letters in the same line are statistically significant at the *p* < 0.05 level according to the Tukey test.

**TABLE 2:** Percentage (%) decrease in microbial viability after direct CAP application to microorganisms.

Microorganism name	Decrease in microbial viability (%)					
	100 W		150 W		200 W	
	30 s	60 s	30 s	60 s	30 s	60 s
*S. aureus* ATCC 25923	8.87	22.10	12.50	34.11	17.92	45.58
*L. monocytogenes* ATCC 7644	9.61	24.71	15.57	36.70	20.09	51.03
*S. typhimurium* CCM 5445	10.85	28.65	17.43	39.88	24.60	54.65
*S. enteritidis* ATCC 13076	11.80	30.07	19.90	41.65	23.70	56.75
*E. coli* O157:H7	13.20	34.50	19.80	46.30	27.10	60.80
*C. albicans* ATCC 10231	6.71	15.10	10.48	21.18	17.63	40.19

To further assess the influence of CAP treatment on microorganisms, the CAP‐treated distilled water samples (at various power levels and times) were mixed with cultures in two different ratios: 1:1 and 1:9. According to the analysis results, in both cases, the viability of bacteria decreased as the applied flow rate and CAP contact time increased. However, microorganisms continued to grow (Tables 3, 4, 5, 6). In CAP distilled water samples combined with bacterial culture at a 1:1 ratio, the smallest decrease in the number of viable cells was observed in *C. albicans* ATCC 10231 with 2.14% (7.59 log CFU/mL) at 100 W and 30 s, while the highest decrease was observed in *E. coli* O157:H7 644 with 60.14% (3.21 log CFU/mL) at 200 W and 300 s (Table 3 and Table 4). Among the microorganisms combined with CAP distilled water at a 1:9 ratio, *E. coli* O157:H7 suffered the most significant reduction (*p* < 0.05) in viable cells, losing 70.26% (2.39 log CFU/mL) after 300 s at 200 W. In contrast, *C. albicans* ATCC 10231 exhibited the smallest decline, with only 4.05% (7.45 log CFU/mL) fewer viable cells observed after 30 s at 100 W (Table 5 and Table 6).

**TABLE 3:** Number of living cells as a result of the application of microorganism + CAP distilled water (1:1) (log CFU/mL).

Microorganism name	100 W				150 W				200 W				Control microorganism log CFU/mL
	Time (s)												
	30 s^a^	60 s^x^	180 s^i^	300 s*	30 s^b^	60 s^y^	180 s^ii^	300 s^#^	30 s^c^	60 s^z^	180 s^iii^	300 s^+^	
*S. aureus* ATCC 25923	7.79 ± 1.5	7.63 ± 1.8	6.95 ± 2.9	6.44 ± 2.0	7.67 ± 1.2	7.57 ± 2.4	6.63 ± 1.4	6.21 ± 1.9	7.56 ± 3.2	7.32 ± 1.0	6.49 ± 1.3	5.45 ± 2.5	8.15 ± 1.4
*L. monocytogenes* ATCC 7644	7.77 ± 1.3^+^	7.49 ± 1.1^+^	6.41 ± 2.4	5.73 ± 1.9	7.52 ± 1.6^+^	6.92 ± 2.0	5.71 ± 1.1	4.85 ± 1.7	7.12 ± 2.5	5.91 ± 1.3	5.01 ± 1.0	4.53 ± 0.9a^,x,b^	8.21 ± 1.7
*S. typhimurium* CCM 5445	7.45 ± 2.0^+^	7.14 ± 1.6	6.22 ± 0.7	5.41 ± 1.1	7.24 ± 2.9^+^	6.63 ± 2.1	5.45 ± 1.6	4.60 ± 1.2	6.84 ± 2.2	5.66 ± 1.8	4.74 ± 1.4	4.21 ± 1.7^a,b^	7.92 ± 1.1
*S. enteritidis* ATCC 13076	7.49 ± 1.7	6.72 ± 1.9	5.56 ± 1.2	5.04 ± 2.6	7.21 ± 2.0	6.03 ± 1.3	4.40 ± 2.1	3.98 ± 1.8	6.73 ± 2.2	5.45 ± 1.4	4.08 ± 2.6	3.32 ± 1.3	8.14 ± 2.5
*E. coli* O157:H7	7.40 ± 1.1^ii,#,iii,+^	6.51 ± 1.4^+^	5.46 ± 2.0	4.84 ± 1.5	7.0 ± 1.8^#,iii,+^	5.78 ± 1.1	4.32 ± 0.8^a^	3.72 ± 1.5^a,b^	6.53 ± 2.2^+^	5.17 ± 1.6	3.88 ± 1.4^a,b^	3.21 ± 1.8^a,b,x,c^	8.05 ± 1.6
*C. albicans* ATCC 10231	7.59 ± 0.8	7.48 ± 1.3	7.36 ± 2.4	6.70 ± 1.9	7.47 ± 3.0	7.37 ± 1.6	7.20 ± 2.1	6.90 ± 1.8	7.38 ± 2.7	7.28 ± 2.3	6.99 ± 1.6	6.70 ± 12	7.76. ± 0.9

*Note:* ^a,b,c x,y,z i,ii,iii,*,#,+^Values expressed with different letters in the same line are statistically significant at the *p* < 0.05 level according to the Tukey test.

**TABLE 4:** Percentage (%) decrease in microbial viability after the application of microorganism + CAP distilled water (1:1).

Microorganism name	Decrease in microbial viability (%)											
	100 W				150 W				200 W			
	30 s	60 s	180 s	300 s	30 s	60 s	180 s	300 s	30 s	60 s	180 s	300 s
*S. aureus* ATCC 25923	4.42	6.35	14.73	20.98	5.87	7.07	18.69	23.76	7.23	10.18	20.35	33.19
*L. monocytogenes* ATCC 7644	5.45	8.78	21.93	30.15	8.36	15.72	30.47	40.89	13.28	27.99	39.05	44.83
*S. typhimurium* CCM 5445	5.96	9.86	22.48	31.75	8.63	16.32	31.13	41.87	13.69	28.51	40.17	46.79
*S. enteritidis* ATCC 13076	7.97	17.48	31.72	38.08	11.48	25.93	45.96	52.62	17.29	33.01	49.88	59.18
*E. coli* O157:H7	8.05	19.08	32.19	39.85	12.55	26.71	46.28	53.75	18.84	35.73	51.79	60.14
*C. albicans* ATCC 10231	2.14	3.58	5.17	13.60	3.71	5.05	7.23	11.02	4.92	6.18	9.87	13.65

**TABLE 5:** Number of living cells as a result of the application of microorganism + CAP distilled water (1:9) (log CFU/mL).

Microorganism name	100 W				150 W				200 W				Control microorganism log CFU/mL
	Time (s)												
	30 s^a^	60 s^x^	180 s^i^	300 s*	30 s^b^	60 s^y^	180 s^ii^	300 s^#^	30 s^c^	60 s^z^	180 s^iii^	300 s^+^	
*S. aureus* ATCC 25923	7.63 ± 2.8^+^	7.23 ± 1.6^+^	6.49 ± 2.9	5.78 ± 1.7	7.49 ± 1.5^+^	6.91 ± 1.0	5.88 ± 14	5.01 ± 1.7	6.18 ± 3.1	6.00 ± 2.0	5.08 ± 1.4	4.15 ± 1.8^a,b,x^	8. 15 ± 1.4
*L. monocytogenes* ATCC 7644	7.64 ± 1.3^#,iii,+^	7.09 ± 1.9^+^	6.09 ± 0.5	5.19 ± 1.0	7.43 ± 2.8^+^	6.62 ± 2.2	5.35 ± 1.5	4.47 ± 1.2^a,b^	6.91 ± 1.3^+^	5.53 ± 1.8^a^	4.65 ± 2.1^a,x,b,c^	3.90 ± 1.9	8.21 ± 1.7
*S. typhimurium* CCM 5445	7.33 ± 1.9^+^	6.70 ± 2.2^+^	5.56 ± 1.4	4.45 ± 1.1	7.12 ± 2.6^#,iii,+^	5.94 ± 2.4	4.81 ± 1.8	3.95 ± 1.2^b^	6.61 ± 1.9	5.18 ± 1.1^+^	4.07 ± 1.3^b^	3.38 ± 2.0^a,x,b,c^	7.92 ± 1.1
*S. enteritidis* ATCC 13076	7.40 ± 2.1^*,iii,+^	6.44 ± 1.7^+^	4.98 ± 2.0	4.40 ± 1.4^a^	7.04 ± 2.8^iii,+^	5.79 ± 2.2^+^	4.53 ± 1.9	4.49 ± 1.2	6.32 ± 1.6^+^	4.90 ± 1.0	3.88 ± 1.7^a,b^	2.77 ± 1.4^a,x,b,y,c^	8.14 ± 2.5
*E. coli* O157:H7	7.23 ± 1.6^*,ii,#,iii,+^	6.02 ± 1.2^+^	4.54 ± 2.8	3.89 ± 2.1^a,b^	6.84 ± 2.3^*,#,iii,+^	5.38 ± 1.4^+^	4.05 ± 1.5^a^	3.43 ± 0.7^a,b^	6.01 ± 1.0^+^	4.58 ± 1.9	3.41 ± 1.6^a,b^	2.39 ± 1.1^a,x,b,y,c^	8.05 ± 1.6
*C. albicans* ATCC 10231	7.45 ± 0.9	6.95 ± 1.4	6.50 ± 2.0	6.16 ± 1.7	7.28 ± 1.2	6.62 ± 1.0	6.10 ± 2.2	5.44 ± 1.6	7.11 ± 1.9	6.28 ± 1.5	5.83 ± 11	5.22 ± 1.5	7.76. ± 0.9

*Note:* ^a,b,c x,y,z i,ii,iii,*,#,+^Values expressed with different letters in the same line are statistically significant at the *p* < 0.05 level according to the Tukey test.

**TABLE 6:** Percentage (%) decrease in microbial viability after the application of microorganism + CAP distilled water (1:9).

Microorganism name	Decrease in microbial viability (%)											
	100 W				150 W				200 W			
	30 s	60 s	180 s	300 s	30 s	60 s	180 s	300 s	30 s	60 s	180 s	300 s
*S. aureus* ATCC 25923	6.38	11.27	20.37	29.03	8.07	15.21	27.90	38.52	11.96	26.33	37.64	49.10
*L. monocytogenes* ATCC 7644	7.00	13.65	25.86	36.80	9.45	19.41	34.88	45.57	15.78	32.63	43.40	52.47
*S. typhimurium* CCM 5445	7.42	15.36	29.81	43.75	10.12	24.95	39.27	50.09	16.62	34.65	48.63	57.38
*S. enteritidis* ATCC 13076	9.12	20.86	38.79	45.99	13.15	28.57	44,29	52.36	22.37	39.82	52.31	66.02
*E. coli* O157:H7	10.23	25.19	43.56	51.73	15.09	33.20	49.71	57.40	25.36	43.08	57.60	70.26
*C. albicans* ATCC 10231	4.05	10.45	16.21	20.60	6.21	14.73	21.44	29.92	8.33	19.11	24.93	32.68

In another method, CAP was applied to a combination of microorganisms + distilled water (1:1 and 1:9) at different power rates and times. In both conditions, no microbial growth was observed at 300 s (except *C. albicans* ATCC 10231), while lower viable cell counts were observed than the control group at 30, 60, and 180 s (Tables 7, 8, 9, 10). Among the microorganisms combined with distilled water (at both 1:1 and 1:9 ratios), *C. albicans* ATCC 10231 displayed the least susceptibility. Its viable cell count decreased by only 3.21% (7.51 log CFU/mL) at 1:1 (Tables 7 and 8) and 5.58% (7.13 log CFU/mL) at 1:9 (Tables 9 and 10) after 30 s at 100 W, respectively. In contrast, the highest decrease was observed in *E. coli* O157:H7. Its viable cell count plummeted by an impressive 65.93% (2.74 log CFU/mL) and 69.72% (2.44 log CFU/mL) after 180 s at 200 W, respectively (*p* < 0.05).

**TABLE 7:** Number of viable cells as a result of CAP application to the combination of microorganism + distilled water (1:1) (log CFU/mL).

Microorganism name	100 W				150 W				200 W				Control microorganism log CFU/mL
	Time (second)												
	30 s^a^	60 s^x^	180^i^	300*	30 s^b^	60 s^y^	180 s^ii^	300 s^#^	30 s^c^	60 s^z^	180 s^iii^	300 s^+^	
*S. aureus* ATCC 25923	7.68 ± 1.8	7.20 ± 1.2	5.77 ± 1.5	—	7.53 ± 1.3	6.96 ± 2.0	5.53 ± 1.1	—	7.04 ± 1.9	6.08 ± 1.2	5.13 ± 1.6	—	8. 15 ± 1.4
*L. monocytogenes* ATCC 7644	7.71 ± 1.3^iii^	7.17 ± 1.1	5.35 ± 1.7	—	7.47 ± 1.6	6.68 ± 1.5	4.88 ± 1.1	—	6.99 ± 2.1	5.71 ± 1.4	4.70 ± 1.0 ^a^	—	8.21 ± 1.7
*S. typhimurium* CCM 5445	7.43 ± 1.0^ii,iii^	6.84 ± 1.8	5.10 ± 1.6	—	7.15 ± 2.2^iii^	6.40 ± 1.6	4.52 ± 1.4 ^a^	—	6.73 ± 1.7	5.18 ± 1.8	4.01 ± 1.3 ^a,b^	—	7.92 ± 1.1
*S. enteritidis* ATCC 13076	7.40 ± 1.5^ii,iii^	6.06 ± 2.3	4.71 ± 1.8	—	7.10 ± 1.2^ii,iii^	5.60 ± 1.6	3.92 ± 1.0 ^a,b^	—	6.54 ± 1.9^iii^	4.55 ± 1.2	3.25 ± 15 ^a,b,c^	—	8.14 ± 2.5
*E. coli* O157:H7	7.26 ± 2.1^i,ii,iii,z^	5.84 ± 1.4 ^iii^	4.35 ± 1.2 ^a^	—	6.81 ± 1.6 ^ii,iii^	4.96 ± 1.3	3.70 ± 0.9 ^a,b^	—	6.44 ± 1.8 ^iii^	4.26 ± 1.3 ^a^	2.74 ± 0.6 ^a,x,b,z^	—	8.05 ± 1.6
*C. albicans* ATCC 10231	7.51 ± 1.6	7.18 ± 1.2	6.27 ± 1.0	5.80 ± 1.6	7.40 ± 1.1	6.97 ± 1.5	5.85 ± 2.0	5.14 ± 1.5	7.20 ± 1.8	6.75 ± 1.4	5.39 ± 1.3	4.63 ± 1.0	7.76. ± 0.9

*Note:* ^a,b,c,x,y,z,i,ii,iii^Values expressed with different letters in the same line are statistically significant at the *p* < 0.05 level according to the Tukey test.

**TABLE 8:** Percentage (%) decrease in microbial viability after CAP application to the combination of microorganism + distilled water (1:1).

Microorganism name	Decrease in microbial viability (%)								
	100 W			150 W			200 W		
	30 s	60 s	180 s	30 s	60 s	180 s	30 s	60 s	180 s
*S. aureus* ATCC 25923	5.78	11.63	29.18	7.63	14.55	32.18	13.65	25.43	37.15
*L. monocytogenes* ATCC 7644	6.05	12.75	34.80	9.02	18.63	40.53	14.87	30.50	42.75
*S. typhimurium* CCM 5445	6.18	13.69	35.60	9.78	19.25	42.89	15.05	36.41	49.38
*S. enteritidis* ATCC 13076	9.03	25.50	42.18	12.79	34.49	51.85	19.73	44.07	60.05
*E. coli* O157:H7	9.86	27.40	45.93	15.41	38.43	54.08	20.06	47.11	65.93
*C. albicans* ATCC 10231	3.21	7.48	19.18	4.68	10.13	24.65	7.21	13.14	30.48

**TABLE 9:** Number of viable cells as a result of CAP application to the combination of microorganism + distilled water (1:9) (log CFU/mL).

Microorganism name	100 W				150 W				200 W				Control microorganism log CFU/mL
	Time (s)												
	30 s^a^	60 s^x^	180^i^	300*	30 s^b^	60 s^y^	180 s^ii^	300 s^#^	30 s^c^	60 s^z^	180 s^iii^	300 s^+^	
*S. aureus* ATCC 25923	7.53 ± 0.8^iii^	7.06 ± 1.4	5.48 ± 1.2	—	7.34 ± 2.2^iii^	6.07 ± 1.3	5.18 ± 1.0	—	6.86 ± 1.8	5.71 ± 1.3	4.42 ± 1.7 ^a,b^	—	8.15 ± 1.4
*L. monocytogenes* ATCC 7644	7.54 ± 1.2^ii,iii^	6.93 ± 1.9	4.96 ± 1.3	—	7.35 ± 1.7 ^iii^	5.96 ± 1.0	4.63 ± 1.5 ^a^	—	6.90 ± 1.3	5.32 ± 0.9	4.18 ± 1.2 ^a,b^	—	8.21 ± 1.7
*S. typhimurium* CCM 5445	7.24 ± 1.9 ^i,ii,iii^	6.42 ± 1.1	4.30 ± 1.6 ^a^	—	7.00 ± 1.5 ^ii^	5.54 ± 1.3	3.87 ± 1.8	—	6.31 ± 1.4 ^a,b^	4.77 ± 1.0	4.24 ± 1.7 ^a^	—	7.92 ± 1.1
*S. enteritidis* ATCC 13076	7.28 ± 1.4 ^i,ii,z,iii^	5.79 ± 0.9	4.11 ± 1.2 ^a^	—	6.89 ± 1.4 ^ii,iii^	4.95 ± 1.1	3.54 ± 1.8 ^a,b^	—	5.98 ± 2.3 ^iii^	4.14 ± 1.6 ^a^	2.93 ± 1.4 ^a,b,z^	—	8.14 ± 2.5
*E. coli* O157:H7	7.13 ± 3.1 ^i,ii,z,iii^	5.63 ± 1.9 ^iii^	3.95 ± 1.6 a	—	6.65 ± 1.3 ^ii,z,iii^	4.75 ± 1.7	3.25 ± 1.1 ^a,b^	—	5.72 ± 1.2 ^iii^	3.78 ± 1.5 ^b^	2.44 ± 1.0 ^a,x,b,c^	—	8.05 ± 1.6
*C. albicans* ATCC 10231	7.33 ± 1.5^+^	6.89 ± 1.1	5.85 ± 2.0	5.33 ± 1.8	7.16 ± 1.2^+^	6.47 ± 1.4	5.36 ± 1.9	4.72 ± 1.3	7.06 ± 1.8^+^	6.19 ± 1.3	5.08 ± 1.4	3.95 ± 1.8 ^a,y,c^	7.76. ± 0.9

*Note:* ^a,b,c,x,y,z,i,ii,iii^Values expressed with different letters in the same line are statistically significant at the *p* < 0.05 level according to the Tukey test.

**TABLE 10:** Percentage (%) decrease in microbial viability after CAP application to the combination of microorganism + distilled water (1:9).

Microorganism name	Decrease in microbial viability (%)								
	100 W			150 W			200 W		
	30 s	60 s	180 s	30 s	60 s	180 s	30 s	60 s	180 s
*S. aureus* ATCC 25923	7.63	13.41	32.78	9.88	25.60	36.48	15.91	30.01	45.77
*L. monocytogenes* ATCC 7644	8.12	15.60	39.53	10.45	27.76	43.55	16.06	35.20	49.06
*S. typhimurium* CCM 5445	8.63	19.02	46.30	11.62	30.10	51.08	20.36	39.82	53.49
*S. enteritidis* ATCC 13076	10.53	28.87	49.57	15.40	39.13	56.45	26.57	49.17	64.10
*E. coli* O157:H7	11.45	30.08	50.94	17.39	41.02	59.63	28.93	53.08	69.72
*C. albicans* ATCC 10231	5.58	11.27	24.61	7.71	16.62	30.90	9.03	20.27	34.60

**FIGURE 5:**
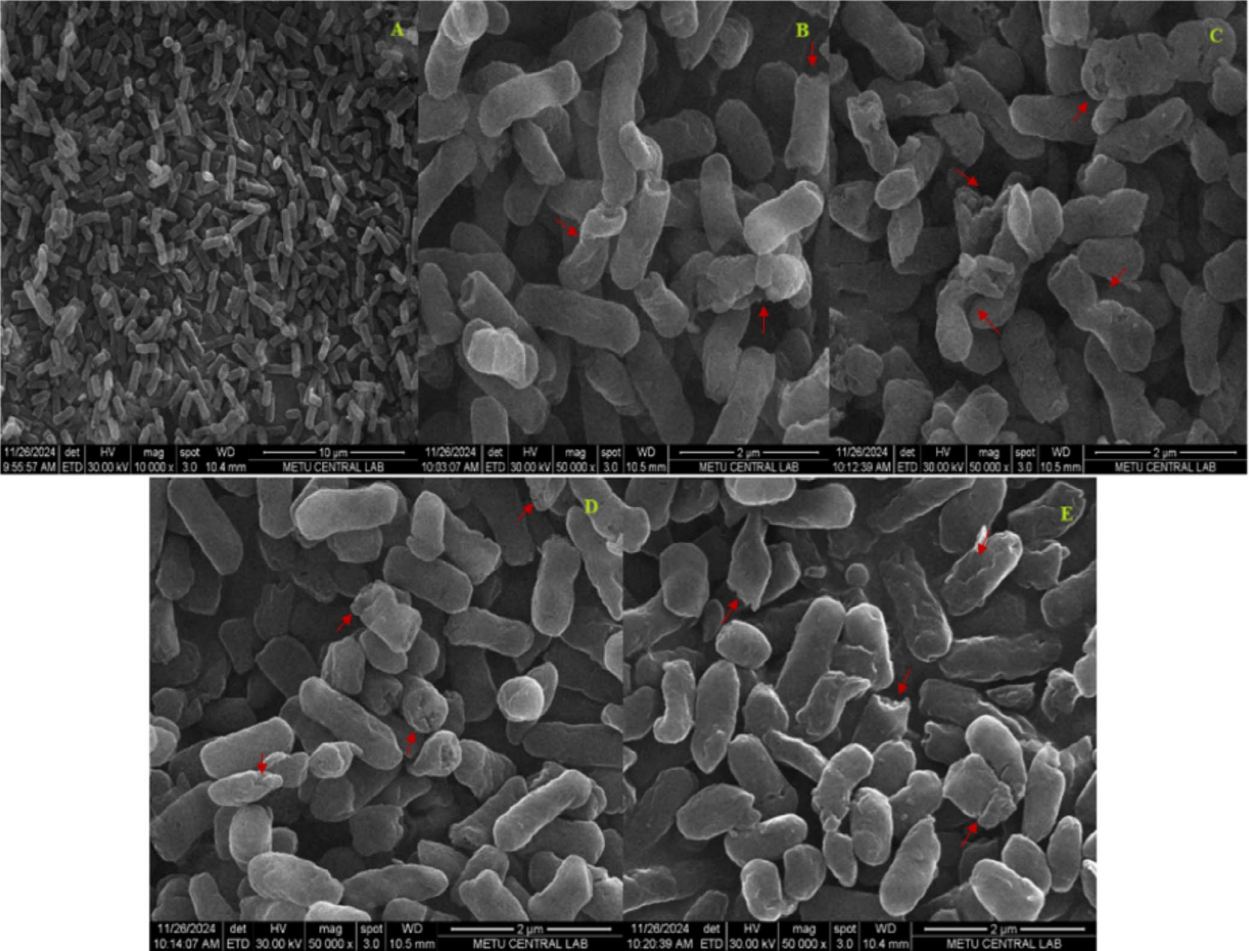
SEM images of *E. coli* O157:H7. Red arrows indicate changes in cell morphology or damage. (a) Non‐treated (b) microorganisms were directly treated with CAP for 60 s (c) microorganisms were directly treated with CAP for 300 s (d) PAW (300 s) e) CAP‐treated microorganisms + distilled water combination (300 s).

To examine the morphological alterations induced by CAP treatment, *E. coli* cells were analyzed using SEM. Following CAP with three different methods (microorganism, distilled water, and distilled water + microorganism combination), bacterial cells exhibited significant morphological damage, including shrinkage, disruption, and pore formation (Figure 5). As the treatment duration increased, the severity of damage became more pronounced. Initially, smooth cell membranes developed small holes and wrinkles, eventually leading to severe deformation and cell rupture. Direct plasma treatment for 300 s resulted in the most severe damage, demonstrating the irreversible and destructive impact of cold plasma on *E. coli* cell membranes.

## 4 Discussion

CAP treatment has the potential to inhibit the growth of pathogenic microorganisms that cause food spoilage and foodborne illness. This could lead to longer shelf lives for food products, without the need for harmful preservatives or chemicals. Research has extensively explored the direct application of CAP and PAW to various food products, including vegetables, fruits, nuts, and powdered foods, focusing on their potential to combat pathogenic microorganisms and increase shelf life (Han et al. 2020b; Moutiq et al. 2020; Wang et al. 2023). Therefore, unlike typical approaches, this study tested the usefulness of three innovative cold plasma methods, targeting the pathogenic microorganisms behind food spoilage and disease rather than treating the food directly.

In the application of CAP, both process parameters (such as gas type and composition, application time, surface properties of the food matrix, relative humidity, plasma application method, and flow rate) and microbial factors (such as bacterial load, substrate, temperature, pH, and composition of the growth medium) play an effective role (Huang et al. 2018; Tappi et al. 2023).

Unlike studies that applied CAP directly to food products, in this study, CAP was applied to foodborne pathogens. This expanded our perspective of the potential of CAP for food safety applications. The study investigated the effectiveness of CAP using three different methods: Microorganism, distilled water, and distilled water + microorganism combination. This approach has the potential to be more effective in inactivating pathogens, as it targets the specific organisms that are causing spoilage or illness.

Kim et al. (2011) applied CAP to sliced pork bacon inoculated with *L. monocytogenes, E. coli*, and *S. typhimurium* . They used two different gas compositions (helium and helium+oxygen), three different powers (75, 100, and 125 W), and two different times (60 and 90 s.). They found that the helium treatment resulted in a 1–2 log decrease in the number of inoculated pathogenic microorganisms, while the helium + oxygen gas mixture treatment resulted in a 2–3 log reduction. In another study, CAP was applied to chicken meat and skin contaminated with *Listeria innocua* . The study found that a 10‐s application resulted in a > 3 log decrease in *L. innocua* , while an 8‐min application in the skin resulted in a 3 log reduction. A 4‐min application also resulted in a 3‐log reduction in the number of *L. innocua* on muscle (Noriega et al. 2011). Yong et al. (2015) inoculated cheese slices with *E. coli, S. typhimurium*, and *L. monocytogenes* and then applied a DBD treatment at 250 W and 15 kHz. They reported a log reduction of 1.65 in the pathogens inoculated on the cheese slices. In another study that investigated the viability of *S. typhimurium* in lettuce, strawberries, and potatoes using cold plasma treatment, a 2.71 log inhibition was seen after 2 min of treatment, and a 2.72, 1.76, and 0.94 log reduction was observed after 15 min of treatment, respectively (Fernández et al. 2013). Matan et al. (2015) examined the effects of green tea extract and atmospheric Radio Frequency plasma (RF) against pathogens (*E. coli, S. typhimurium*, and *L. monocytogenes* ) on fresh‐cut dragon fruit. They reported that 60 s of 40 W RF plasma treatment with 5% green tea inhibited all pathogens on the surface of fresh‐cut dragon fruit. In contrast, the researchers reported that when fresh‐cut dragon fruit was treated with only 2.5%–10.0% green tea without plasma treatment, not all bacterial growth on the surface could be prevented.

Prasad et al. (2017) spot‐inoculated tomatoes with *E. coli* and then applied CAP treatment for 5, 10, 15, or 30 min. at 15 and 60 kV. The researchers reported that CAP treatment at 60 kV for 15 min. resulted in the highest inhibition of *E. coli* on the tomatoes. In another study that investigated the inactivation of *E. coli* ATCC 700891 in mandarin juice using high‐voltage electrical discharge plasma, the sample was treated at various voltages (17–30 kV), discharge frequencies (20–80 Hz), and temperatures (15°C–35°C). According to the study, at the most appropriate factors (30 kV, 40 Hz, and 25°C), a 2‐min plasma treatment resulted in a 4.8 log CFU/mL reduction in *E. coli* . In a study by Moutiq et al. (2020), chicken samples were treated with CAP at 100 kV for 1, 3, and 5 min. The researchers found that within 5 min of treatment and 24 h of storage, the natural microbiota of the chicken meat was reduced by approximately 2 log CFU/g. Another study investigated the effectiveness of cold plasma in inhibiting common foodborne bacteria from Gram‐positive ( *L. monocytogenes* and *Bacillus cereus* ) and Gram‐negative ( *Salmonella typhi* and *Vibrio cholerae* ) groups. Starting with a high concentration of bacteria (1.5 × 10^8^ CFU/mL), they examined how long the bacteria could survive when exposed to the plasma. After 10 min of treatment at 25 kV, the population of *S. typhi, V. cholerae, B*. *cereus* , and *L. monocytogenes* was significantly reduced to 0.3 × 10^2^, 1.5 × 10^2^, 0.6 × 10^3^, and 0.7 × 10^5^ CFU/mL, respectively (Khatami et al. 2023).

Frías et al. (2020) investigated the effectiveness of CAP for the inactivation of *Salmonella enterica* serovar Enteritidis, *S. enterica* serovar Typhimurium, *L. monocytogenes* , and *E. coli* O157:H7 on tofu. The researchers found that a 15‐min CAP treatment resulted in a log10 inhibition of 0.2–0.6 for *S. enterica* serovar Enteritidis and *E. coli* O157:H7, respectively. The researchers also investigated the potential of PAW as a dipping solution to control microbial growth on tofu during its shelf life. They detected that storing tofu in the refrigerator using PAW efficiently controlled microbial growth. A study investigating the inhibition effect of PAW against *Pseudomonas deceptionensis* CM2 isolated from spoiled chicken meat reported that *P. deceptionensis* CM2 was decreased by approximately 5 log units within 10 min of exposure to PAW (Xiang et al. 2018). In a recent study by Wang et al. (2023), researchers created PAW by sparking electric discharge beneath the surface of sterile distilled water. They then studied the properties of PAW, focusing on its effectiveness in inhibiting bacteria and how it works. They used *E. coli* , a common foodborne pathogen, as their target organism. They found that PAW progressively killed more *E. coli* as the discharge and immersion times increased. The greatest reduction, reaching 4.25 log CFU/mL (a massive decrease in the number of bacteria), was achieved. In another study, the effectiveness of PAW against *Enterobacter aerogenes* was investigated, and a 1.92 log CFU/mL inhibition of *E. aerogenes* was seen with PAW (Joshi et al. 2018).

Previous studies have established that Gram (+) bacteria exhibit higher resistance to CAP treatment compared to Gram (−) bacteria. This difference stems from variations in their cell wall structures. Gram (+) bacteria typically possess a thicker, multi‐layered cell wall with complex peptidoglycan layers compared to the thinner wall of Gram (−) bacteria. This strong wall acts as a shield, hampering the diffusion of reactive species produced by cold plasma and compromising its effectiveness (Fröhling et al. 2012; Ziuzina et al. 2014; Han et al. 2020b). This study, employing Gram (+) and Gram (−) species, confirmed this approach. We observed that Gram (+) bacteria consistently displayed higher resistance across all three applied CAP methods, exhibiting lower inhibition rates compared to Gram (−) bacteria.

Ding et al. (2023) examined the inactivation efficacy of cold plasma against *Alicyclobacillus acidoterrestris* . SEM analysis demonstrated that cold plasma treatment induced significant damage to the cell membrane, with the severity of damage increasing with treatment time (0, 15, 30, and 60 s). A separate study employed SEM to assess the morphological alterations induced by plasma treatment in *Bacillus subtilis* cells. The results indicated that CAP application caused significant damage to the cell membrane (Charoux et al. 2020). In their study, Zhou et al. (2019) employed SEM to examine the morphological alterations in *Botrytis cinerea* mycelium following CAP treatment. They observed that untreated *B. cinerea* mycelium had an intact structure covered with filamentous polysaccharides, while treated mycelium exhibited folding and collapse. Our results are largely in agreement with these studies.

## 5 Conclusion

Unlike other studies in which CAP was applied, our study aimed to prolong product shelf life by preventing pathogens' development before adverse effects such as decay and spoilage are observed in foods. In the study, three different CAP applications (direct application to microorganisms, distilled water (PAW), and a combination of bacteria and distilled water) were used for inhibiting pathogens. All three applications reduced the viability of *S. aureus* , *L. monocytogenes*, *S. typhimurium* , *S. enteridis*, *E. coli* , and *C. albicans* . In particular, it was observed that CAP treatment applied directly to microorganisms reduces viability in a short time or completely inhibits the bacteria. The fact that significant reductions in the viability of pathogens were detected with CAP application to water suggests that it could be used effectively in agricultural applications in the fight against pests that cause diseases in plant roots and in plant cultivation (increasing seed germination and growth, root, stem, and leaf growth, and increasing plant resistance). CAP is a promising novel technology for the inactivation of foodborne pathogens. All three CAP treatments used in the study have the potential to be used as antimicrobial agents to combat pathogens in different areas. CAP treatment of food products could be beneficial for keeping food fresh and preserving it for longer without spoilage. This research has made substantial contributions to the field by thoroughly exploring the ability of CAP systems to inhibit foodborne pathogens. It has successfully shown that effective sterilization can be achieved at low temperatures through the use of various methods. This research provides a new direction for future research, underscoring the potential of CAP systems for improving food safety.

## Author Contributions

**Berat Cinar Acar:** conceptualization, investigation, writing – original draft, methodology, supervision, project administration.

## Ethics Statement

As the article does not contain any studies with human or animal subjects, its approval by the ethics committee was not required.

## Conflicts of Interest

The author declares no conflicts of interest.

## Data Availability

The data that support the findings of this study are available from the corresponding author upon reasonable request.
